# Laparoscopic pyeloplasty in pediatric patients: The SGPGI experience

**DOI:** 10.4103/0970-1591.60441

**Published:** 2010

**Authors:** Ruchir Maheshwari, M. S. Ansari, Anil Mandhani, Aneesh Srivastava, Rakesh Kapoor

**Affiliations:** Department of Urology and Renal Transplantation, Sanjay Gandhi Postgraduate Institute of Medical Sciences, India

**Keywords:** Laparoscopic pyeloplasty, pelvi-ureteric junction obstruction, pediatric patients

## Abstract

**Objectives:**

To determine the safety, efficacy and long-term outcome of laparoscopic pyeloplasty in pediatric patients.

**Materials and Methods:**

A prospective analysis of data of pediatric patients under the age of 15 years, who had undergone laparoscopic pyeloplasty from January 2000 to June 2008 was done. The various parameters analyzed were; operative time, blood loss, need for analgesics, intra/postoperative complications, hospital stay and postoperative outcome. Success was defined on the basis of either improvement in the symptoms/or better drainage on postoperative isotope renography.

**Results:**

A total of 82 patients with a mean age of 7.12 years (four months to 15 years) and male to female ratio of 4.3:1 were included in the study. Dismembered pyeloplasty was done in 70 patients and Foley Y-V plasty in 12 patients via transperitoneal approach using three ports in 79 or four ports in three children. Mean operative time was 151 minutes (78-369); mean blood loss was 88.01 ml (50-250) with a mean hospital stay of 5.05 days (2-11). Conversion to open surgery was required in four (4.87%) patients. Follow-up renograms were available in 74 patients who showed improvement in drainage in 69 patients and obstructed pattern in five; of these two patients had significant deterioration in split function. Two patients among the obstructed group underwent redo pyeloplasty by open technique while the rest three elected for conservative approach. At a mean follow-up of 41.58 months (8-75) the overall success rate was 91.89%.

**Conclusion:**

Laparoscopic pyeloplasty is effective and safe in children with minimal morbidity and gives excellent long-term results.

## INTRODUCTION

Pelvi-ureteric junction obstruction (PUJO) is one of the most common causes of obstructive uropathy in children. Minimally invasive surgical alternatives like balloon retrograde dilation and endopyelotomy were described to avoid the attendant morbidity of open procedure, but were plagued with low success rates.[1–4] Conventional open Anderson Hynes dismembered pyeloplasty remains the gold standard surgical treatment with a long term success rate exceeding more than 90%.[5–9]

Laparoscopic pyeloplasty (LP) is well described in adults and has the same success rate as open with significantly less morbidity and complications.[10] Feasibility of LP in children was described with similar success rates as open procedure, but experience remains limited.[11–13] We present our experience of laparoscopic pyeloplasty in children.

## MATERIALS AND METHODS

This is a prospective analysis of 82 pediatric patients under the age of 15 years who had undergone laparoscopic pyeloplasty in the period from January 2000 to June 2008. The demographic data such as patient characteristics, operative time, blood loss, need for analgesics, intra/ postoperative complications, hospital stay and postoperative outcome were analyzed. Preoperative evaluation included an ultrasonography, renal function tests, urine culture and intravenous urography (IVU). Functional assessment of renal function and obstruction were done by a diuretic renogram in all the cases. This included a well-tempered diuretic renogram as proposed by international consensus committee and approved by Society of Nuclear Medicine.[14] Ultrasound done at our institute (demonstrating dilated renal pelvis with normal/non-visualized ureter) along with dynamic renal scan were used to confirm diagnosis in case of any doubts. Indications of intervention included either documentation of an obstruction on renogram, or symptomatic patient with hydronephrosis on IVU. Renal moiety with split function ≥10% was taken for pyeloplasty. Pelvi-ureteric junction obstruction (PUJO) was primary in all patients. Secondary stones were present in five patients. First follow-up renogram was performed at three months post surgery with subsequent scans at three-monthly intervals in first year and then at six-monthly intervals for the next two years, as per our institutional protocol. All cases were done by transperitoneal approach.

All the children received bisacodyl rectal suppository the night before surgery to ensure that colon was empty. Intraoperative broad-spectrum antibiotic was given to all. Under general anesthesia, an adequate size Foleys catheter was inserted and left on free drainage during the surgery. RGP was not done routinely. Preoperative JJ stent was put retrogradely in 13 patients. This practice was changed later and, in the remaining of the patients, JJ stent was placed antegradely during the surgery itself.[15] In one patient JJ stent could not be placed and a ureteric catheter was left.

All children were operated via transperitoneal approach with the child placed in flank position. Forty seven cases were on left side and 35 on right side. The three ports were used most often and the fourth was rarely inserted for the purpose of retraction. It was required in three cases on right side for retraction of liver. The usual port placement included; a 3/5/10-mm camera port was put just lateral to the umbilicus by open technique, in children aged less than two years, two to 10 years and more than 10 years of age respectively. Intra-abdominal pressure was kept at 8-10/10-12/12-14 mm of Hg in respective age groups. Two additional ports; one each of 3 or 5 mm working ports, were placed in subcostal and at spino-umbilical line [Figure 1a]. (Ethicon 5 mm and 10 mm ports, Tyco Versaport 5-11 mm, Karl Storz 3 and 5 mm reusable ports). We used Karl Storz 3 mm, 5 mm and 10 mm 30° telescopes, Karl Storz L-hook electrode, dissector, grasper and suction (of size 3 mm and 5 mm), Ethicon 5 mm needle holder and Ethicon 5 mm Harmonic scalpel. Colon was reflected medially, renal pelvis and upper ureter were dissected free from the surrounding tissues. Crossing vessel [Figure 1b], when identified, was carefully dissected from the renal pelvis for transposition.

**Figure 1a:**
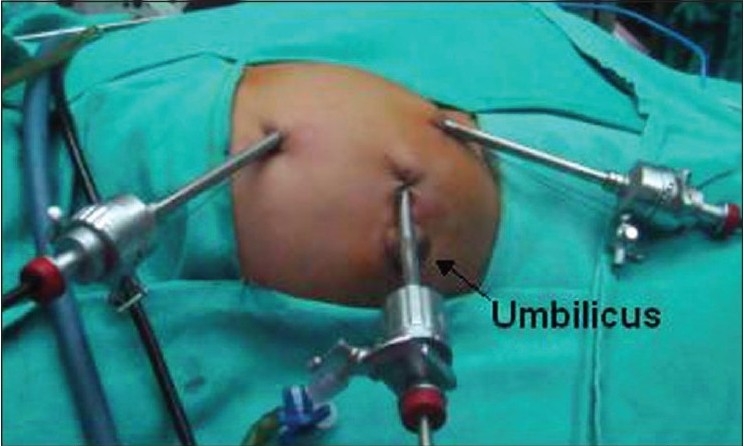
Port placement for laparoscopic pyeloplasty

**Figure 1b:**
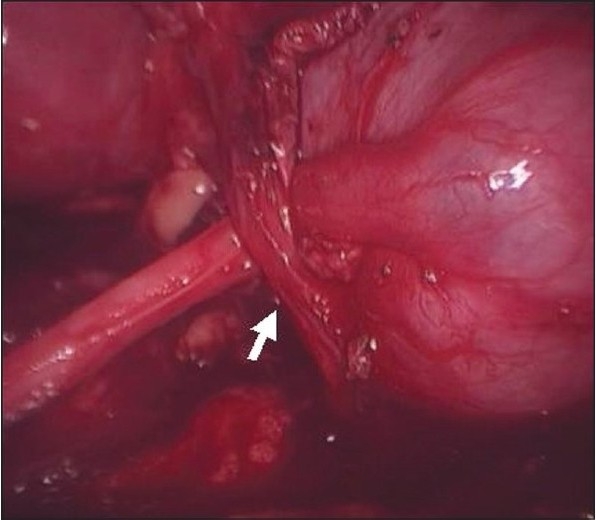
Anterior crossing vessel in a child with right side pelvi-ureteric junction obstruction

Pelvis was cut medially, from above downwards, hence preparing the lower lip of the pelvis for anastomosis. The ureter was spatulated laterally. Three-suture technique was used for uretero pelvic anastomosis at most dependant part of the cut renal pelvis; [10] 5-0 vicryl was used to place the first suture at the apex of the spatulated ureter and this suture was later used for continuous suturing of the posterior anastomotic line. Another 5-0 vicryl was used to place the second suture at the cut end of ureter and the corresponding site in renal pelvis that is later used for continuous suturing of the anterior anastomotic line. The third continuously running suture was used for the closure of the remaining renal pelvis. A trans-anastomotic JJ ureteric stent was placed in ante grade manner [Figure 2a]; if not already placed retrogradely; over a guide wire via subcostal port after posterior layer was sutured. The stiff end of a guide wire was passed through a 5 Fr ureteral catheter and then through the open end of a Double-J stent to straighten the close end of the stent. Artery forceps were applied to hold the assembly. It was passed through a 5 mm instrument (laparoscopic hook or suction cannula) and the stent was allowed to project 1 cm ahead of the tip of the laparoscopic hook. A rubber cap was applied to the proximal end of the 5 mm instrument to prevent gas leakage.[15] PUJ repair was not retroperitonalized. Abdominal tube drain was put in all the patients through one of the 3/5-mm working ports.

**Figure 2a:**
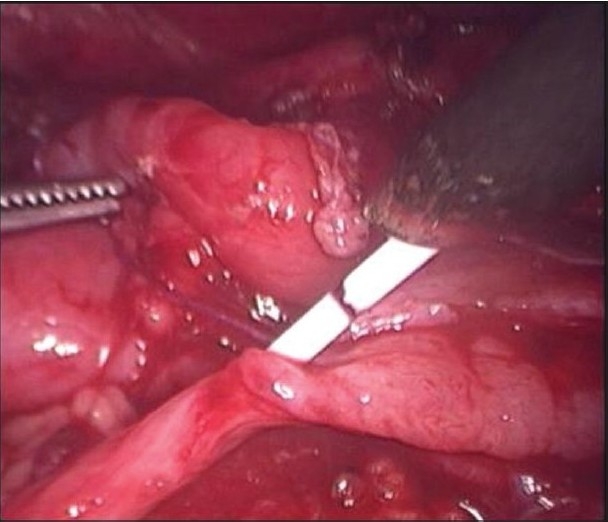
Antegrade stenting during laparoscopic pyeloplasty

A (3 or 5 mm) non-traumatic grasping forceps was used for the stone extraction. Stones were extracted from the kidney and placed in a pouch made from cut thumb of surgical glove. The opening of the pouch was clipped and subsequently removed at the end of the procedure from umbilical port. Stone clearance was ensured by intraoperative fluroscopy.

## RESULTS

Eighty-two children with a mean age of 7.12 years (four months-15 years) and male to female ratio of 4.3:1 had undergone LP. All the patients had primary PUJ obstruction and five had secondary renal stones. Dismembered pyeloplasty was done in 70 patients and Foley Y-V plasty in 12 patients. Double J stent was put before the procedure retrogradely in 13 patients while in 64 it was placed during the procedure antegradely. Stentless pyeloplasty was done in four patients as no internal stent was placed in these patients [Table 1].

**Table 1:** Demographic details

Parameter	Value
Age	7.12 years (4 month - 15 years)
Male: Female	4.3:1
Procedures performed	
Dismembered pyeloplasty	70
Foleys Y - V plasty	12
Double J stent placement	
Retrograde	13
Antegrade	64
Stentless pyeloplasty	4

Crossing vessel was found in seven (8.5%) cases. The mean operative time was 151 minutes (78-369 minutes) with a mean blood loss of 88.01 ml (50-250 ml) and mean hospital stay of 4.85 days (2-11 days). While in patients with secondary stones the mean operative time was 198 minutes (120-300 minutes) with a mean blood loss of 148 ml (100-200 ml) and mean hospital stay of four days (3-7 days). Conversion to open surgery was required in four (4.87%) patients [Table 2]. Reasons for conversion were; perinephric adhesion and poor progression (2), lost calculi (1) and lost suturing needle (1). The stone was large, of 8 mm in longest diameter. However, small stones can be left. The needle was large, of 3-0 suture in the first case. In the second one, we used 5-0 suture which has tiny needle. The situation was informed to the parents of the child also and shared decision was taken to leave needle *in situ*.

**Table 2:** Perioperative details

Parameters	Mean	Range
Operative time	151 min	78-369 min
Blood loss	88.01 ml	50-250 ml
Hospital stay	4.85 days	2-11 days
Conversion	4 (4.87%)	
Analgesic use	4 days	3-5 days

None of the patients required blood transfusions. For pain control paracetamol was used in the dosage of 15 mg/kg/dose, which was required for a mean duration of four days (three to five days).

Most of the complications were minor and were managed conservatively while major complications required intervention [Table 3]. Most of the complications were encountered in the early stage of the study as the occurrence of these declined with the time and experience [Figure 2b].

**Figure 2b:**
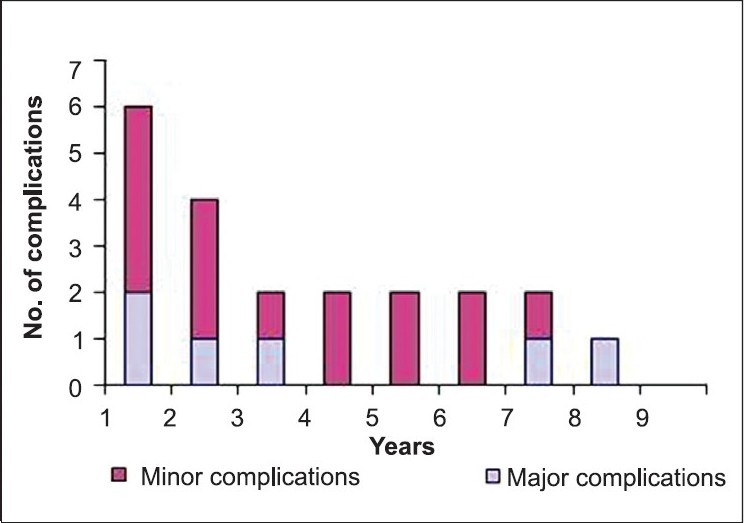
Year-wise display of complications encountered

**Table 3:** Complications

Minor complications	
High drain output	5
Postoperative ileus	4
Fever	3
Postoperative adhesive acute intestinal obstruction	1
Port site hernia	1
External urethral meatus stenosis	1
	
Major complications	
Anastomotic leakage	2
Lost suturing needle	2
Omental prolapsed through drain site	1
Urinary peritonitis	1

Follow-up renograms were available for 74 patients which showed improvement in drainage in 69 patients and obstructed pattern in five; of these split function improved in 64 patients while in eight it remained stable and deteriorated significantly in two. The mean split renal function was 35.37% (range 12-55%) and 42.5% (range 9-70%) (*P* < 0.05) before and after surgery respectively.

Renogram showed obstructed pattern in five; of these two patients had significant deterioration in split function. Cases with gross hydronephrosis were associated with failure. Stenosis was found at the site of anastomosis during re-exploration; there was a possibility of ischemic injury. Two patients among the obstructed group underwent redo pyeloplasty by open technique while the rest three elected for conservative approach. Of these three cases who opted for conservative management, two had gross hydronephrosis with dependent lower pole. There was no failure in stentless pyeloplasty. Thus at a mean follow-up of 41.58 months (8-75), overall success rate was 91.89%.

## DISCUSSION

Open dismembered pyeloplasty has been the standard of care for the treatment of PUJO with reported success rate in excess of 90%.[5–9] Laparoscopic pyeloplasty is less invasive and well established in adults with reported success rate comparable to open pyeloplasty.[10] There have been successful reports of LP in children but it still remains to be an established procedure until large series with long term outcome are available.[11,17–19] The present series of 82 children is the second largest prospective series of LP to the best of our knowledge.[20]

The most commonly described access remains the transperitoneal although retroperitoneal approach has also been reported; both the approaches have their own pros and cons.[17–25] We used transperitoneal access in all patients. Placement of stent in LP; ante grade verses retrograde, still remains a matter of debate.[15–19,23] Cystoscopic DJ stenting adds to operating time and may not be hassle free in smaller children. In the present study DJ stent was placed retrogradely in 13 children till the new technique of antegrade stenting was described in 2002[15] and since then it was placed in ante grade manner in 64 patients successfully. Difficulty in placing DJ stent was encountered in one patient in whom a 4 Fr. ureteric catheter was placed retrogradely and removed after 24 hours. Stentless pyeloplasty was done in four patients. Of these, two patients had high drain output for more than five days; one developed perinephric collection and low grade fever which necessitated DJ stenting on fifth postoperative day while in one it subsided gradually on its own.

Crossing vessel was found in only seven (8.5%) children in the present series. This incidence is significantly lower than the other reported series. Janetschek *et al*.,[26] have shown that crossing vessel was present in 35% (16/331) of the normal unobstructed renal units and this incidence was two times higher in obstructed renal units. Inagaki *et al*., [27] reported 54% (80/145 units) incidence of crossing vessel in their patients managed by LP. Reason for such a low incidence of crossing vessel in the present series is unexplainable.

The overall mean estimated blood loss, mean operative time and mean hospital stay of this series are comparable to other series in children.[19–23] In a recently published study of transperitoeal LP in children, 28 out of 29 were completed successfully and mean operative time was 255 min (range 157-396), mean estimated blood loss 10 ml in all.[21] Similarly, in other studies of transperitoneal LP in children, 16 out of 16 were completed successfully and mean operative time was 160 min (range 90-270), mean estimated blood loss was 60 ml (range not given) and mean hospital stay was not reported,[19] mean operating time was 219 min (range 140-310) with a mean hospital stay of 2.4 days (range 1-5) and 2/22 (9%) required conversion to open surgery.[23] Majority of the cases with higher blood loss were during the initial part of the series (learning curve effect). The mean was also affected by the cases with associated renal stones, in which there was higher blood loss.

The conversion rate in the present series (4.87%) is comparable to other published series in children (0-9%)[19–23] and adults (0-5.4%).[26,28–30] Although no major intraoperative complication was noted, the overall postoperative complication rate in the present series was 17.94%, which is comparable to the other pediatric series of LP (15-18.7%).[19–23] Most of the complications in our series were minor and managed conservatively. Further, most of the complications were encountered in early phase of the study and there was a linear decline in the rate of complications with the buildup of experience [Figure 2b].

It is worth mentioning that the learning curve for LP in children is more steep and long compared to their adult counterparts. We say this on the basis that at our center we first tried the procedure on adults; only after obtaining satisfactory results we started this procedure for pediatric patients.[15,16] Besides, the LP procedure is a little more difficult and tricky in children under five years of age as compared to older ones due to the small size of abdomen providing lesser intraperitoneal space to work. Mean duration of follow-up in the present series is 41.58 (8-75months), which is the longest reported mean follow-up for pediatric LP to the best of our knowledge. Overall success rate of 91.89% is well comparable to other pediatric series, which range between 87-100%.[17,19–23]

## CONCLUSION

Data on LP in pediatric patients is still limited as compared to adults as long-term prospective series are yet to come. LP is effective and safe in children with minimal morbidity and gives excellent long-term results.
